# Book Reviews

**Published:** 1824-10

**Authors:** 


### CRITICAL ANALYSIS

OF

### ENGLISH AND FOREIGN LITERATURE

RELATIVE TO THE VARIOUS BRANCHES OF

### Medical Science.

Quæ laudanda forent, et quæ culpanda, vicissim

Illa, prius, cretâ; mox hæc, carbone, *notamus*.—PERSIUS.

### DIVISION I.

### ENGLISH.

ART. I.—*A Practical Treatise on Diseases of the Skin, comprehending an Account of such Facts as have been recorded on these Subjects; with original Observations. The whole arranged with a View to illustrate the Constitutional Causes of these Diseases, as well as their local Characters.* By SAMUEL PLUMBE, Member of the Royal College of Surgeons of London, of the Medico-Chirurgical Society, &c. &c.—8vo. pp. 392. London: Underwood, 1824.

THIS volume bears a very close resemblance, in most of its features, to the well-known and justly celebrated work of Dr. BATEMAN. Like it, it is an octavo of above three hundred pages. It is entitled a "Practical Treatise on Diseases of the Skin," being as close a synonym of Dr. Bateman's "Practical Synopsis of Cutaneous Diseases," as the English language admits of. Like it, too, it is embellished with a frontispiece, representing the characters of the principal orders of cutaneous diseases. The designations of these diseases are, with scarcely any exception, the same as those of WILLAN; and it is plain that the one work is intended to supersede the other. The first inquiry, then, which naturally suggests itself to our minds is, what new or improved views of the subject have been taken by Mr. PLUMBE, to justify us in discarding our old guides? what alterations in theory or practice has he made? and how far has he succeeded in correcting old errors, or enlarging the bounds of prior knowledge. Dr. Bateman's work must be acknowledged by all as one of infinite merit. There are few of us who have not had opportunities of verifying the accuracy of his descriptions. For copiousness and correctness, indeed, they have long appeared to us to be unrivalled. Nay, we had even looked upon many of them as unsusceptible of improvement; and it was with no small anxiety that we turned over Mr. Plumbe's pages, to ascertain how he had contrived to give a new view of the subject, without copying from Willan and Bateman on the one hand, or sacrificing fidelity of description on the other.

It will not be considered by the public as detracting from the merits of the book, nor, we would hope, by the author as derogating from his dignity, when we say that, in this dilemma, he has chosen the right path; that he has freely acknowledged his obligations to these authors, and, in many instances, (as in pages 160, 170, 230, 238, 240, 257, 274,) copied largely and avowedly from them. In what circumstances, then, consists the difference between the rival works of Plumbe and Bateman? Is it in their views of practice? Has Mr. Plumbe discovered any new methods of combating these intractable diseases, for which we have so often (and so often in vain) turned over Dr. Bateman's pages? or is it only that he shows us our old friends in new dresses? We trust that our readers have not stretched their expectations too far, for we fear it will be found that this latter is the true answer to our question. We are far from thinking, however, that this necessarily involves a censure of the book. If Mr. Plumbe has, in his new views of an old subject, improved our notions of the relative bearings of its different parts, and given greater consistency and totality to the whole,—if he has made what was before obscure, intelligible,—if he has lopped off redundancies, and opened up the avenues which led to the obscure and hitherto inaccessible parts of the science, we, in our humble estimate of the march of human knowledge, think he has done a great deal. Now this is what we conscientiously think Mr. Plumbe has done; and we feel, therefore, as members of the profession, under obligations to him for the pains he has taken in investigating, with so much pathological acumen, the different forms of chronic cutaneous diseases, and for the skill he has displayed in their arrangement. We shall give the best proof we can of our sincerity in this acknowledgment, by appropriating an unusually large portion of our pages to the analysis of the work. We shall take the liberty of interspersing occasionally a few critical remarks; and, as we like to get the most disagreeable part of our duty over as soon as possible, we shall begin by a few comments on Mr. Plumbe's *style.* In perusing his book, we remarked, with regret, numerous instances of utter inattention to all the rules of literary composition. We do not expect fine writing in a work purely professional, but we do expect, and we think not without reason, perspicuity, precision, and, above all, correctness of grammatical construction. The following instances, selected from among several which we had noted for animadversion, will satisfy our readers (and, we venture to think, the author himself,) that the next edition will be susceptible of some improvement.

"It has been already remarked, that the pustules *are uniformly having* a hair growing in their centre." (Page 51.)—"On the other hand, however, it must be acknowledged, that we *are*

*often seeing* them co-existent on the same head." (P. 55.)—"Such principle is abused *at the same time as it is* partially carried into effect." (P. 54.)—"Where two *importantly different diseases* are confounded together." (P. 57.)—"Whatever may be the opinion of the profession generally as to the necessity of a more *minutely local* attention to cutaneous diseases." (P. 69.)—In page 46, in speaking of cleanliness and good nurses, we are recommended to "a diligent use of the *domestic materials* such *descriptions* of persons are accustomed to employ in washing the children's heads." The last sentence of page 62, and the second of page 45, are so much involved, as to prevent the meaning from being obvious on the first perusal. In page 35, speaking of frictions, the author says, "*a few days under its use* have disclosed pretty considerable collections of matter;" which, though intelligible, is very inelegant.—"The ring-worm of the scalp may be identified with a cutaneous disease, *commonly similarly* named." (P. 46.)

These, and various other instances which might be produced of careless and faulty writing, are real and serious drawbacks on the merit of the book. They would for ever prevent the sentences, or passages, in which they occur, from being quoted in other works; and we strongly urge the author, on this as well as on other accounts, to a careful revision of his language. It is with a view of drawing his attention to the subject, that we have made these criticisms; in the hope of seeing a good book freed, in a future edition, from blemishes so easily removed.

Mr. Plumbe commences with some remarks on the anatomy and physiology of the skin. He speaks at some length of the origin and growth of the hair; and, as he is particularly desirous of pressing this question upon the attention of his readers, we shall commence our extracts by quoting the passage.

"The cutis, therefore, a vascular and highly sensible structure, is penetrated by the hair, which does not, as far as the most minute injections, with the help of glasses, can show, receive any contribution of vessels for the purposes of nourishment from it: and if there is any analogy between the structure of the human hair and that of the larger species of animals, the oleaginous secretion covering it, and giving to it its smooth and glossy appearance, is also derived from the adipose structure alluded to beneath the cutis; this fluid being conveyed along the centre of the hair by tubes which originate in the bulb." (P. 8.)

The scalp, then, according to Mr. Plumbe, is pierced by the hair, but has little or no share in its production or nourishment. This doctrine he afterwards applies in determining the causes of that affection, which he considers the most obstinate of all cutaneous diseases, not having a constitutional origin or connexion,—viz. ringworm of the scalp.

Another point of cutaneous anatomy, which the author insists on, as calculated to explain cutaneous pathology, may also be given in his own words:—

"The cutis, on the vascular energies of which the production and nourishment of the cuticle depends, besides its more obvious and general offices as a covering to other parts, and as the structure effecting the separation of the perspirable matter from the mass of blood, gives, in different parts of the body, a seat to a most important structural arrangement, on the disorder of which some of the most obstinate cutaneous affections are found to depend,—the sebaceous follicles. These follicles are minute thimble-like depressions in the substance of the cutis. The larger kind are most numerously distributed over parts much exposed, and where flexures of the skin are formed: the secretion poured out in the former instance probably forming a defence to the cuticle, under exposure to heat; and in the latter, operating to prevent the consequences of attrition. They are most easily distinguishable about the nose and mouth in men, as well as in females; but, in the latter, they are also often seen in great numbers on the neck and upper parts of the chest: their secretion, which is entirely supplied by the vessels of the cutis, gives an agreeably smooth and glossy appearance to the skin of these parts, where their dimensions and number are not very considerable; but, where the reverse is the case, the secretion at their orifices becomes discoloured, forming so many minute black spots, which much disfigures these parts, and gives them a dingy, unhealthy appearance." (P. 3, 4.)

Mr. Plumbe is deeply impressed with the importance of distinguishing chronic cutaneous diseases, according as they are *local* or *constitutional;* and the following is the arrangement which, in conformity to his views on this point, he has adopted. He divides cutaneous diseases strictly so called, (for he, with great propriety, refuses to admit the exanthemata into his classification,) into five sections. The *first* includes such as are *local,* and obtain their distinguishing characters from local peculiarities of the skin: they are Acne, Sycosis, and some species of Porrigo. The *second* embraces those which are constitutional, the derangement of the system being of the nature of *debility,* with consequent diminished tone of the vessels of the cutis. This section includes Purpura and Scurvy, Pemphigus and Pompholyx, Ecthyma and Rupia. Mr. Plumbe comprises in his *third* division all such cutaneous diseases as are constitutional in their origin, and in their progress are characterised by active inflammation. He further states it as his opinion, that the derangement of the system producing these diseases, exists primarily and mainly in the digestive organs; and he adds, that they probably exert a salutary influence on the body. These two latter characters are evidently much too theoretical to be admitted as groundworks of a nosological arrangement. The diseases included in this order are Porrigo Favosa and Larvalis,

Strophulus, Lichen, Prurigo, Urticaria, Herpes, the Thrush, and Boils. The *fourth* section is appropriated to diseases marked by chronic inflammatory action of the vessels producing cuticle,—viz. Lepra, Psoriasis, Pityriasis, Pellagra, and Icthyosis; the constitutional causes, or influence, being here uncertain. The *fifth* and last of Mr. Plumbe's sections is made to comprise such diseases as are of a mixed character, depending essentially on active cutaneous inflammation, with which, however, (derangement of) the constitution is not, as in the diseases of the third section, *necessarily* connected. In this section the following diseases are associated together: Impetigo, Scabies, and Eczema. In a sort of Appendix, we meet with some remarks on symptomatic cutaneous inflammations of trifling importance, viz. Erythema and Roseola, on the characters of Venereal Eruptions, on Lupus, and Porrigo decalvans.

Such is Mr. Plumbe's arrangement of chronic cutaneous diseases; and we think our readers will acknowledge it to be possessed of considerable merit. We are not sure whether the different sections follow each other in a strictly natural order. It has occurred to us that, if they were transposed in the following manner—1, 4, 3, 5, 2, the succession would be much more readily understood, and the appearance of a *system* better preserved. We are, upon the whole, however, well satisfied with the author's classification; and we think it but a small compliment to him to say, that it is decidedly superior to the well-known, but most unscientific, division of Drs. Willan and Bateman.

We shall now take up in detail each of the subjects treated by Mr. Plumbe, laying stress upon those passages which appear to us interesting, either on account of the novelty or the pathological or practical importance of the opinions advanced.

Section 1. *Acne*.—This disease consists essentially, in its original form, of simple obstruction to the free passage of the sebaceous matter to the surface of the skin, in consequence of which that substance accumulates and hardens. The necessary result of such accumulation, in course of time, is the distension of the follicles in which it is contained, and their consequent inflammation. Mr. Plumbe does not think, as was believed by Willan and Bateman, that such inflammation, once excited, ever terminates in resolution. Independent of the disposition to this affection which habit of living may generate, some individuals are peculiarly exposed to it from original formation of skin; and hence has arisen the idea of its hereditary character. When disorder of the digestive organs has been the immediate exciting cause of acne, the symptoms of the first affection (dyspepsia) are alleviated, and more formidable mischief sometimes prevented by its occurrence. So at least Mr. Plumbe thinks; but we are very doubtful of the correctness of this opinion. Suppuration is the most desirable course acne can take. Repellents, as they have been termed, seldom have any effect in preventing it. Frequent bathing the parts with warm water, and gentle friction with the mildest kind of soap "constitutes" (constitute) by far the best kind of local application. The notion of blistering for the removal of acne, originated with Dr. Darwin. Mr. Plumbe has no doubt of its immediate effect in stopping the disease, by clearing the mouths of the obstructed follicles; but it is a remedy unnecessarily severe, and exceedingly inconvenient. The carbonate of soda, and the oxymuriatic acid, are useless, unless coupled with attention to what Mr. Plumbe calls the local treatment *surgically.*

"The foregoing remarks apply more particularly to the states and consequences of obstruction of the sebaceous follicles, denominated acne simplex and acne punctata. When the disease occurs more extensively, and habitual disorder of the digestive organs or scrofulous diathesis exist, a slow and unhealthy suppuration takes place, spreading from the originally inflamed follicle, and involving to a considerable extent those in the neighbourhood; producing, instead of a remote pustule, a considerable, though slowly formed, collection of matter. The latter, instead of finding its way quickly to the surface, accumulates, and disorders the substance of the cutis to a great extent. Its course and extent is marked, during the more active state of the inflammation, by a florid looking and very irregularly formed, rather prominent tubercle, exceedingly tender to the touch, and in some one or two points soft: still the superincumbent skin is not ruptured, and in the course of a short time it assumes a dark-blue colour. In this state it sometimes comes under the notice of the surgeon; when, the existence of matter being ascertained, a lancet is thrust into it, and the contents discharged, leaving for some time an unhealthy livid edge to the orifice, which slowly heals, and, in most instances, leaves a mark of some duration on the part." (P. 26, 27.)

In this state of disease, the identity of which with simple acne may be satisfactorily proved, local stimulants, prior to the discharge of the matter from the tubercle, are highly improper. But, after this, a lotion, composed of five grains of corrosive muriate, dissolved in eight ounces of proof spirit, may be advantageously employed. In severe cases, mercurial ointment answers the purpose far better than any thing else. Constitutionally, a tonic plan of treatment is called for.

Sometimes the mischiefs of follicular obstruction and inflammation are confined to the tip of the nose (acne rosacea): of all deviations from health, this obtains the least commiseration. The general impression, that it depends on good living and hard drinking, is a correct one. If the same causes continue to operate, the irregular tuberculated nose is at length formed, and follicular organization is completely destroyed. Emollient applications are here useful. Friction by means of soft brushes, assisted by soap and warm water, are decidedly beneficial.

2. *Sycosis.*—This is nothing more than acne, or follicular obstruction, occurring on parts covered by hair. It is generally true, that affections of such portions of skin as are covered with hair, are comparatively obstinate and formidable; the irritation of the hair aggravating the original disease. The pulling out of single hairs, where it can be done without pain, is often advisable; as thereby irritation is removed, and orifices are formed, by which the contents of a pustule can readily be discharged. Emollient applications are then to be trusted to; no more good being to be expected from sedative washes or stimulants, than in the treatment of the simpler forms of acne.

3. *Porrigo scutulata*, or ringworm of the scalp.—Mr. Plumbe has paid particular attention to this complaint; and we think this decidedly the best chapter in the book. We shall begin by laying before our readers a sketch of Mr. Plumbe's notions concerning the nature and varieties of porrigo. He draws a broad line of distinction between the porrigo scutulata and favosa, believing the former to be altogether a local, and the latter a constitutional, affection; differing essentially from each other in their phenomena, progress, and mode of treatment. It would certainly have been better if, with this impression so strong upon his mind, the author had applied some different term to the former disease; and, for our own parts, we are strongly inclined to think that, in this piece of pathology, Mr. Plumbe is perfectly correct. The true ringworm of the scalp, porrigo scutulata, is defined by him to be an inflammation of a specific character, affecting the solid structure of the cutis, dependent for its support on the operation of locally irritating causes; a tedious and obstinate disease, which destroys the hair; spreads by contagion; is seldom, if ever, known to terminate of its own accord, and difficult of cure, even under the most scientific management. Porrigo favosa, on the other hand, has always a constitutional origin; it frequently terminates spontaneously, and always requires the exhibition of remedies, through the medium of the constitution. Differing from each of these is that state of the scalp designated by Willan *porrigo decalvans.* Here no vestige of disease is ever discoverable in the cutis. In strict pathology, this affection is nothing more than the result of a particular structure ceasing to perform its office.

Such are the three legitimate species of porrigo. We are here to confine our attention to the porrigo scutulata. In its early stage it is called ringworm of the scalp; in the latter, scald head. Dr. UNDERWOOD, according to our author, is incorrect in asserting that the disease commences in the roots of the hair; and Dr. Willan, in stating that minute straw-coloured pustules exist from the first, and are necessary to constitute the disease. Mr. Plumbe has observed that, when pustules are noticed, they are uniformly found with hairs growing through them; and, though he is not prepared to deny the possibility of the occurrence of pustules where no hairs exist, yet experience bears him out in the opinion, that they are produced by the irritation which the presence of hair in an already diseased skin is calculated to excite. The leading feature of true ring-worm of the scalp is the falling-off of the hair; and the cause of this is the excessive excitement of the cuticular vessels, which deprives the structure secreting the hair of their due nourishment. The very same principle explains the desquamation of cuticle taking place after scarlatina. In some instances, this goes on to the separation of the hair; but, if examined closely, it will not be found that the rounded, healthy bulbs are separated also: the same thing takes place in true ringworm. Hence it is, also, that, in the early stage of this affection, a scurfy state of the scalp is generally met with; the vessels secreting the cuticle performing their office with a similarly excited action.

Ringworm originates in the application of an infectious matter to a portion of the scalp, and afterwards spreads by the secretion of the pustules. This fact Mr. Plumbe considers highly important in reference to treatment; for it is clear, he says, that "all applications to the part, whether sedative or stimulant, astringent or otherwise, if they do not possess the power of taking from the matter secreted by decomposition, or other means, the power of infection, must be utterly inefficacious, if not (as many kinds of them certainly are) highly prejudicial.

*Treatment of ringworm.*—The first advice of the author is to remove any hair remaining on the affected portion of the scalp which may appear to come away easily, and without pain to the patient; after which the part is to be shaved. The next step, preliminary to any medical application, is to effect the discharge of as many of the pustules as possible, by pinching up the skin between the finger and thumb, and carefully washing away what is thus forced out with warm water and soap. The third step in the treatment is to employ some astringent application, possessing the power of taking from the secretion its infectious properties.

"A solution of the sulphate of copper has been employed in some cases for this purpose: I believe the object to be more completely accomplished, however, by rubbing this preparation, in a finely powdered state, on the part, and then washing it off." (P. 72, 73.)

A careful examination should be instituted every morning, and, if any pustules appear, they should be at once removed, and the sulphate of copper applied, as at first, to the part. Where the disease is severe, six weeks or two months will be required for its cure. Mr. Plumbe sets himself to oppose vigorously the sweeping anathema pronounced by Willan against depilation. Drawing out the hair does not destroy the structure secreting it; and, however barbarous the means by which it is effected, the removal of the hair is never followed by baldness. It is unnecessary to say that the author objects strongly to the pitch-cap, which, tearing off alike the sound and the unsound hair, and the scabby secretions of a tender surface, inflicts a degree of pain, from which "imagination shrinks with horror:" but from these inconveniences the use of a pair of forceps is perfectly free.

The only other peculiarity in Mr. Plumbe's treatment of ringworm, is the application, in very severe and obstinate cases, of adhesive straps and bandages. In one very obstinate case, (described page 80,) they completely succeeded; and, though the constant irritation of the newly-springing hair occasioned difficulties which were considered insurmountable, still the author had eventually the satisfaction of seeing the part completely covered with long and glossy hair.

In cases where there is no actual formation of pustule, a morbid redness of the scalp, and a production of scurf, sometimes remain. To remove this, the author recommends, in the first instance, the assiduous application of a spirituous lotion, with frequent washing of the scalp. Where it has been of long duration, a weak solution of lunar caustic is very beneficial, alternately with the former.

"In condemning the use of greasy applications of any kind, which have not the power of destroying the infectious properties of the contents of the pustules, I am justified, not only by reasoning *a priori*, but by the evidence of facts; for I have frequently seen the disease spread with greater activity after such applications, than before they were made use of.

"The necessity of internal remedies in cases of simple ringworm, or that stage of the disease usually seen in the middle and higher classes, where attention to the frequent cleaning of the children's heads is observed, may with much propriety be doubted. It is certain that, in such classes, we rarely trace the slightest connexion of the disease with constitutional causes; but, under different circumstances, as in cases approaching in similarity to that last alluded to, the occurrence of severe constitutional irritation may be expected, and a feverish state of the system is rarely absent. Constitutional treatment is, of course, then directed, to subdue symptoms consequent on the irritation of the disease, and not to correct a state of the system, on which it may be supposed to depend." (P. 82, 83.)

Porrigo *furfurans* and porrigo *lupinosa* are next spoken of; and, being regarded as varieties of the P. scutulata, the same principles are applied to them.

Section II. *Purpura.*—Forty-four pages of Mr. Plumbe's work are occupied with a discussion concerning the pathology of purpura, the object of the author being to prove "that this formidable disease, whatever may have been its predisposing cause, is immediately brought about by obstruction in the hepatic circulation, and consequent impediment to the functions of the stomach and alimentary canal." In this opinion the author may be right, or he may be wrong; but we cannot understand how he makes out purpura to be a cutaneous disease, after such an avowal; more particularly as, in page 372, he refuses to admit into his arrangement certain diseases, "which obtain their chief importance from their connexion with, and dependence upon, constitutional disturbance, and the treatment of which is solely directed by the existing constitutional symptoms."

All that we shall say of the reasonings contained in this chapter is, that it has failed to convince us of the accuracy of the opinions advocated. Among other points, the author proposes the following abstruse question; and afterwards offers a solution, which appears to us by no means satisfactory.

"Is the highest degree of hepatic and general visceral congestion and obstruction in the abdomen, with which we are acquainted, capable of so impeding the functions of digestion and chylification, as to become a cause of such reduction in the nutrient properties of the blood, as to render this fluid unequal to the efficient nourishment of every part of the system? If it is, in what parts of that system would the debility consequent thereon be first manifested?" (P. 125.)

"With respect to the first of these questions, if a positive answer cannot be readily given in the affirmative, it is at least to be considered not improbable. For the second, it will occur to us, that parts already built up by the previous healthy action of vessels, and not dependent on the latter every hour for their vitality,—parts in a state of quietude and rest, are not those in which such debility would be expected first to appear. The vessels themselves in ceaseless action, and constantly under the influence of a distending power, would, reasoning on common principles, of necessity, be the first to suffer. The vasa vasorum supply the coats of the vessels themselves no better than the latter supply other parts; and these, therefore, being called on to make greater efforts in resisting the impulse of the circulation, first disclose the general deficiency by the rupture of their extremities." (P. 125, 126.)

2. *Pemphigus and Pompholyx.*—Mr. Plumbe agrees with Bateman in the non-existence of true pemphigus. Pompholyx sometimes makes its appearance as an epidemic; three recent instances of which are collected by the author, one occurring in

France, and two in England. The pemphigus infantilis of Willan wears the character of a very aggravated state of pompholyx, with great debility and low fever.

Mr. Plumbe, in his description of this disease, makes considerable use of the Memoir concerning it by Dr. Stokes, published in the 19th volume of this Journal.

3. *Ecthyma and Rupia.*—These are pustular diseases, occurring, for the most part, in young men, who, with constitutions originally not of the strongest class, imprudently indulge in excesses and irregularities to a very great extent, accompanied by privation of rest, and other depressing circumstances. Ecthyma is frequently mistaken for a venereal eruption, but mercury aggravates the disease. Measles, scarlatina, and other diseases, may become exciting causes of ecthyma. Under such circumstances, the eruption is seen, in its very earliest stage, about the waist. Rupia has been satisfactorily shown, in two cases which have fallen under the author's notice, to be only a higher grade of ecthyma; the scab becoming gradually elevated, and a broader ring of growth being added to its base every three or four days.

Anxious to do every justice to Mr. Plumbe's work, we shall take this opportunity of stating that, throughout the whole of it, he has carefully abstained from multiplying names, and subdividing diseases into species differing from each other in points of no pathological importance. The consistence, colour, and structure of scabs, says he, are only the consequences of neglect; and yet, in descriptions of cutaneous disease, they have often obtained much more consideration than the state of the skin, the accumulated secretions of which compose them. If authors, instead of describing such accidental variations, and founding upon them artificial distinctions, had taken the trouble to *clear them away*, and institute a minute examination of the skin itself, they would have been much more usefully employed. There appears to us much good sense, and a complete emancipation from scientific frippery, in this candid avowal of a disciple of Alibert and Willan.

Section III. *Porrigo Favosa.*—This is the first in Mr. Plumbe's series of diseases "which exert a probably salutary influence on the system, which are originally produced by, and usually symptomatic of, deranged digestive organs, and which are characterised by active inflammation. The author's description of porrigo favosa is taken (we believe) from Bateman. His remarks on the constitutional treatment necessary in this state of disease, are rather defective. They are in these words:

"In the management of this disease, there are few points of importance, beyond those which are comprehended in attention to the general health. The state of system under which it usually occurs, as a spontaneous disease, will be found more frequently to indicate the necessity for depletion and alteratives, rather than tonics, which have been recommended." (P. 163.)

To make up for this, we have a very full account of the local treatment, which varies, according as the disease is situated on the scalp, or on some other part of the body. In severe cases affecting the scalp (frequently, but not quite correctly, called scalled head,) fomentations and poultices are necessary to subdue the inflammatory action of the vessels of the part; and, when this has been effected "*a little*, attention to the general health is *sometimes* all that is necessary to the cure." It must be obvious to our readers, that Mr. Plumbe does not express himself here with the same decision as when treating of his favourite subject, ringworm.

When the effusion of viscid fluid on the scalp continues obstinate, the author imagines this to depend on a morbidly relaxed state of its vessels; and an effectual application for this will be found in a solution of caustic, (a scruple to the ounce,) or of sulphate of copper, (two drachms to the ounce,) with which the abraded surface may be pencilled two or three times a-day, until the discharge ceases.

Porrigo favosa, like ringworm, spreads rapidly by contagion through families of children: separation of them, therefore, becomes a matter of prudence.

Mr. Plumbe's account of porrigo larvalis is chiefly taken from Dr. Smith's edition of Willan's Works, and he gives a very complete view of the disease. It is characterised by rapid pustulation and excessive discharge. He looks upon it as a good instance of that grand and powerful remedial measure of nature—counter-irritation; the exercise of which is never more necessary than in the constitution of an over-fed infant. It is not to be confounded with those diseases identified with original debility of constitution. Its influence on the system, with reference to hydrocephalus, is a matter of serious consideration. Mr. Plumbe thinks it more than probable that, in this point of view, its beneficial effects far exceed any thing which art can supply, and that death is averted by it in numberless instances. (P. 177.) Satisfied, as we are, that a gross habit of body is that in which crusta lactea chiefly prevails, we think the author has greatly over-rated its beneficial influence, in respect to determinations to the head. The pathology of hydrocephalus and of porrigo appear to us to be altogether different.

"It has been considered an extraordinary circumstance, that, even in the worst cases of P. larvalis, where the disease has extended over the whole scalp and face, no marks or seams of the skin should remain on the part after recovery: an attentive observation of the pathology of the disease, however, fully explains this,—as the discharge is only poured out from the mouths of the irritated vessels on the surface, without the production of ulcerative absorption. When the cuticle is first elevated and broken by the pustule underneath, copious discharge takes place, not only on the particular point which the latter occupied, but the vessels surrounding it partake of the diseased action, and a more extensive surface of secretion is thus produced: were this not the case, the quantity of discharge would be considerably more limited." (P. 179.)

2. *Strophulus.*—We observe nothing in this chapter peculiarly deserving of note, if we except a passage in page 187, where the author favours us with his views concerning the formation of a *pimple.* "It is," says he, "produced by a minute escape of lymph from a distended vessel, and not by an enlargement of an original part of the cutis; or, in other words, of a papilla, as hitherto supposed." We doubt the accuracy of this statement.

3. *Lichen.*—Like strophulus, the milder cases of this affection may be considered indicative of pretty good general health, though it is stated now and then to occur under states of great constitutional debility, when the pimples wear an appearance in colour not much unlike petechiæ. When any accidental cause of disorder of the digestive organs, or exposure to cold, has taken place, the eruption has the characters of an exanthematous fever. The duration of the disease is very uncertain: it sometimes continues four or five weeks, without any material change in its appearance. Suddenly repelling it, is always succeeded by violent disorder of the constitution. The author fails, however, we think, in showing that lichen exerts any positively salutary influence on the system.

"From some observations which I have lately had an opportunity of making on the sulphur-vapour bath, I am induced to think it a most powerful instrument, in the hands of a judicious medical man, in the treatment of lichen, though it should not be recommended till the bowels have been some time kept open, and the system, if the patient be of a full habit, has been a little reduced. The itching and tingling during its operation is rather severe; but it is followed by a much more tranquil state of the circulation in the cutaneous vessels, and the cure is altogether materially expedited by it." (P. 196.)

4. *Prurigo.*—This affection is characterised by the violent itching of papulæ. In its external characters it resembles lichen; but, in the majority of cases, it is constituted of action of a chronic, rather than an active, character. Pimples are by no means necessary to constitute the disease called prurigo, mere itching existing only in a great number of cases. Its similarity to simple lichen justifies some doubts whether any real difference exists between them. Prurigo is, in general, partial; the generative organs and the back being the most usual of its seats. Neglect of ablution is a frequent source of it. Cleanliness and the warm bath are the most important remedial measures, when it occurs on the superior parts of the body. Purgatives (where the strength of the constitution admits of them) should be freely used. Sulphur has no particular advantage over other medicines. The Harrogate and sulphur-vapour baths are commended. Willan's notion that prurigo is produced by a peculiar insect, is not confirmed. In cases of prurigo ani, Mr. Plumbe recommends the black wash. In very old cases, a strong solution of hydr. oxym. is advisable, until the skin acquires a blush of deep red, with heat and smarting. Pencilling with caustic is another effectual mode of removing that tormenting itching which attends the semi-organized pimples of obstinate prurigo.

5. *Urticaria.*—This chapter is full, but we do not observe any thing peculiar in it. Besides, the subject is pretty generally understood.

6. *Herpes.*—This disease, whether appearing under its regular form (H. zoster), or its irregular form (H. phlyctænodes), is materially, as well at its commencement as in its progress, unlike any other cutaneous affection; and its being confounded with eczema, impetigo, or erysipelas, could only result from great inattention. The nature and character of the constitutional causes of herpes are involved in the greatest obscurity.

"In the absence of any data regarding the manner in which disorder of vital organs can produce cutaneous eruptions, we are fully warranted in supposing, from the symptoms usually preceding herpes, and commonly disappearing after this eruption has gone through its course, that mischiefs of some importance to such organs are averted by it: in short, that it exerts all the influence, and is entitled to the estimation of, a natural counter-irritant. In this character it has, in two or three instances, in delicate females, within my own observation, checked the progress of symptoms, which had given rise to great anxiety respecting the state of the thoracic viscera." (P. 249.)

The following is a good specimen of Mr. Plumbe's talent at pathological research.

"The grand distinguishing feature of herpes, namely, the limited size of the vesicles, seems to depend on the cuticle being bound down to the cutis by the adhesive inflammation, during that state of the vessels of the skin, marked by great heat and redness, which precedes actual effusion of serum. Thus, when effusion takes place, it does not elevate the cuticle generally and extensively, as in erysipelas or pompholyx, but only on the precise spot occupied by the mouths of the vessels, from which the fluid escapes. Hence the pearl-like elevated form of the vesicle, and the obviously thinned and distended state of the cuticle forming its parietes." (P. 254.)

Mr. Plumbe next enters on the consideration of the local varieties of herpes, and is full upon the causes and diagnosis of

H. præputialis. We pass over this, however, as not strictly belonging to the consideration of cutaneous diseases.

7. *Aphtha, or Thrush.*—The author distrusts the general opinion, that this disease is vesicular. He has never been able to see it in the form of distinct vesicles, and he is disposed to consider it as more nearly allied in its nature to strophulus. Pimples of strophulus are often, nay generally, visible on the skin of other parts where the thrush makes its appearance. When a child affected by thrush produces disease in the nipple of its nurse, it is absurd to think of changing the nurse. The better plan is to wean the child at once. If the existence of thrush be known sufficiently early, the application of warm water to the nipple after each suckling, would effectually prevent disease appearing in it. When it has taken place, quietude of the part, with a weak saturnine spirituous lotion, will quickly heal it.

The aphtha of adults is a distinctly vesicular disease. It always occurs in low and debilitated states of the system, and is nearly allied to purpura. By the way, we do not exactly understand how the author reconciles the opinions stated at the top of page 273, with what he says at the bottom of page 124.

8. The last in the series of constitutional inflammatory cutaneous diseases, is *furunculus*, or *boil*. There are two species of this affection: the first involving the cutis and cellular tissue beneath it, and often to so great an extent as to conceal the circumstance of its cutaneous origin; the second, vulgarly termed the *blind boil*, is strictly confined to the substance of the cutis. The former usually occupies the arms, thighs, and nates; the latter occurs chiefly on the abdomen, and trunk generally. The same causes produce the two species; there are no pathological differences between them. Young persons of full plethoric habits, enjoying good health and good living, are most subject to them. The author does not speak, from his own experience, of the value of any particular line of treatment in this furuncular diathesis.

Section IV.—Diseases marked by chronic inflammation of the vessels secreting the cuticle; constitutional causes or influence uncertain.

1. *Lepra.*—The pathological features of this disease are sufficiently distinct and defined, but they unfortunately lead to no practical inferences. The author is unable to throw any light on its causes, (or if, with our author, we must use a hard word where a plain one would answer every purpose,) on its *etiology*. Leprous affections are frequently observed in young females in highly respectable circumstances in life, and prove exceedingly unmanageable. "In the majority of such instances," says the author, "an hereditary origin is, I believe, easily ascertained."

Deficient energy in the vessels of the skin, and consequent inadequacy to the production of healthy cuticle, may, he thinks, be fairly presumed to exist in this disease. Tonic medicines, however, with the exception of hydr. oxym., do not seem to be possessed of much power.

2. *Psoriasis.*—We observe nothing in this chapter to remark upon as new or valuable, if we except the detail of a very interesting case, treated at Mr. GREEN's establishment for sulphur-vapour baths, in Bury-street. We have understood, however, from Mr. Green, who likewise is a scientific man, that he was rather disposed to regard the case as an instance of very severe impetigo. A complete recovery followed the use of the sulphureous fumigations, which were employed daily for seven weeks. The arsenical solution, and the sulphur-vapour bath, promise to become instruments of pretty uniform power in the cure of leprous affections. Speaking of the efficacy of ablution and friction, the author, in page 307, thus expresses himself:—"There are opinions on record, indeed, that these measures deserve the consideration of certain remedies in many cutaneous diseases." We have attempted in vain to discover the true meaning of this passage: we apprehend there is some mistake in it.

3. *Pityriasis.*—Scurfy exfoliation indicates debilitated action of the vessels secreting the cuticle. The causes of this debility are primarily constitutional, when bark, good living, and cold sea-bathing, are indicated. Where local debility remains, a strong spirit lotion, holding some acetate of zinc in solution, is very serviceable. Neglect of this complaint sometimes lays the foundation of confirmed porrigo.

4. *Pellagra.*—Mr. Plumbe's account of this disease is abstracted from Dr. HOLLAND's paper in the Medico-Chirurgical Transactions.

5. *Icthyosis.*—In several cases of this deformity, hereditary origin has been distinctly traced. The combined influence of pressure, applied by means of adhesive straps, and of cold lotions, effected a cure in two instances which fell under the author's notice.

Section V.—Diseases of a mixed character.

1. *Impetigo.*—This disease, in different cases and different stages, exhibits vesicles, pustules, and regularly-formed scales. The grand and predominant features of it are extreme irritation and inflammatory action, extensive pustulation, and scabbing; it is succeeded by a proportionate degree of relaxation of the vessels of the part involved. The author is inclined to think that, in by far the larger portion of cases, it may be referred to local causes; but he acknowledges that it is sometimes preceded by disorder of the whole system, and even that an hereditary predisposition to it exists. In the treatment of impetigo, Mr. Plumbe commends, as indispensable, frequent ablutions, which, with some simple alteratives, and the rejection of all ointments, will often subdue it. He speaks with much confidence of the value of a lotion, consisting of three drachms of hydrocyanic acid, seven ounces and a half of distilled water, and half an ounce of spirit of wine. The sulphur-vapour bath also comes in for its share of praise.

2. *Scabies.*—" It is now pretty well understood that itch is a disorder of the skin, entirely dependent on the habits and operations of an insect, to which Providence has allotted this part of the body for occupation and subsistence." Dr. GALES, physician of the Hospital St. Louis, at Paris, has succeeded repeatedly in producing the disease, by confining the insect in his own skin. The author prefers sulphureous fumigations to every other mode of exhibiting that useful, but unfashionable, remedy. At least, we judge so from the manner in which he speaks of it.

" Dr. Horn, of Berlin, and Dr. De Carro, of Vienna, appear to have been next to Dr. Gales in the use of the bath. Subsequently to these, Mr. Wallace, of Dublin, has published his observations; and step by step, by the joint improvements or suggestions of the observers, the instrument, from having been inconvenient and uncomfortable to the patient, is now become not only an important and decided remedy of great value in many cutaneous diseases, but an absolute luxury as regards the patient's feelings." (P. 357.)

3. *Eczema.*—This form of cutaneous disease is characterised by a diffused eruption of vesicles, without inflammatory bases. It has for its local causes the direct rays of the sun, and for its constitutional causes the irritation of mercury in habits peculiarly predisposed.

We do not observe any thing in this chapter, more than we were familiar with from the works of Bateman and others; and we shall, therefore, here take our leave of Mr. Plumbe.

We trust he will pardon us for the liberty we have taken, in the discharge of our critical duties (as we venture to term them), of animadverting upon those parts of his work which appear to us to detract from its merit. We have said enough, we think, to satisfy our readers, that it is a good book, and worthy of a place in their libraries. It does credit to the author's industry and talent; and we can conscientiously say, we have derived much instruction and pleasure from the task, which we have now brought to a conclusion.

Art. II.—*A Practical Treatise on Hæmorrhoids, or Piles, Strictures, and other important Diseases of the Rectum and Anus: being, with some Additions, a Treatise, to which the Jacksonian Prize was adjudged by the Royal College of Surgeons.* By George Calvert, Member of the Royal College of Surgeons in London, and of the Medico-Chirurgical Society, &c.—8vo. pp. 359. London: Callow and Wilson. 1824.

No branch of surgery has been cultivated with more success, within the last few years, than diseases of the rectum and anus; and yet, in this investigation, the advantage gained bears but a small proportion to the number of Treatises written, and the many labourers who have appeared in the field. It unfortunately happens in our profession, that the majority of books are written by the young, and read by the old, practitioners; which is exactly the reverse of what might be expected and desired: hence the same materials are perpetually employed and misapplied, which the more experienced workman would reject as useless; and thus, though he would render his work less bulky, it would be more solid and lasting. To speak more plainly, we deprecate the publication of Jacksonian prizes generally, as prize Essays. They may be very creditable to the talents of the young aspirant; they may display great anatomical knowledge, extensive reading, and intellectual acumen; but they must be deficient in the essential point of *experience*. In detailing the conflicting and opposite opinions of authors of celebrity, they cannot be expected to place any disputed point of practice upon a firmer basis; and, therefore, although amply deserving of the reward they solicit, they can add nothing to our actual knowledge, and do not contribute to the progress of the science in any degree: nay, they do worse; for they too often actually unsettle and throw a doubt over points that were previously pretty well established.

In prefixing these remarks to our account of Mr. Calvert's work, we intend no disrespect to him: indeed, we have rather been anxious to give our honest opinion upon the present occasion. His Treatise is more practical than prize Essays are usually found to be, and therefore less exposed to the objections we have urged above: nevertheless, his book is of a very formidable length, the principal part of it being made up of the reasonings and opinions of the most eminent authors, both English and foreign; whilst the really practical part lies in a comparatively narrow compass, affording, in this respect, a striking contrast to the little Treatise of Mr. Copeland, which, in the space of a few pages, contains a mass of important matter; and to the more recent publication of Mr. White, which, independently of the cases, occupies only eighty-four or eightyfive pages. We are aware that this is no fault of Mr. Calvert's; that the very nature of a prize Essay renders it necessary for the candidate to display all his reasoning, and to bring out all his elementary knowledge: and this brings us back again to the objection we previously made as to the publication of such works. It is, however, but justice to our author to say, that he has throughout evinced an intimate acquaintance with anatomy, a very extensive knowledge of books, and considerable literary attainments.

A book upon Hemorrhoids is sure to attract attention: the disease is of such common occurrence; the distress and inconvenience produced by it are so incessant, so lasting, and so constantly liable to aggravation from year to year; and the reluctance which the patient feels to communicate his sufferings, from the situation of the complaint, altogether render such publications much sought for, and consequently popular. It is the more necessary, therefore, that correct notions be entertained of the nature of the complaint, and some positive conclusions deduced as to the best mode of treating it.

In the first part of this inquiry, we think Mr. Calvert has been very happy in his recommendation of curative means: if he has not discriminated so well as we think might have been done, he has, at least, omitted nothing that has been said or recommended by authors in general. Our author commences by an account of the origin and general character of hemorrhoids, and then goes on to describe the more common forms of these tumors, which he distinguishes, very justly, from the mere varicose condition of the hemorrhoidal veins; a disease comparatively rare, and which is not often, when it exists, the subject of much uneasiness to the patient, unless combined (as it very often is) with the common hemorrhoidal tumor. An accurate description of the formation and growth of these tumors is then given. We need scarcely say that our author disproves the notion formerly entertained, that they were mere dilatations of the veins. He describes the bleedings that sometimes take place from their surface; but he does not, we conceive, dwell strongly enough upon the occasional severity and extent of these bleedings, and which constitute a very principal argument for the performance of the operation by ligature, in preference to incision, under certain circumstances. We have seen more than once, bleeding take place from a vessel of very formidable size, and to an amount which, if frequently repeated and long continued, would have endangered the patient's safety. It is likewise well known that, in more than one recent instance, after the excision of the tumor has been performed, death from hemorrhage has ensued.

We shall here beg leave to quote the description of these tumors, as given by M. De Larroque:—

"If we divide one of these tubercles in the centre, we find a homogeneous parenchyma, very often of a reddish colour, but which sometimes becomes rather white when it is washed in water, and particularly when macerated. If, previous to washing it, we press the tissue, pure bloody serum, or else a very limpid serous fluid, is forced out, as from a sponge.

"It should be remarked that, even in cases where there are varicose veins, this cellular parenchyma is never wanting; so true it is, that to its development the formation of hemorrhoidal tumors must be attributed. In general, wherever veins are discovered, they are placed between the exterior and this organized tissue, and are lost in very minute ramifications. This general disposition of the veins is an additional proof that these hemorrhoidal tumors do not proceed from varices; for in that case they would be found distributed in the body of the tumors, and not upon their surface." (P. 33.)

Our author adds, that the great difference in the appearance of these tumors in the living and dead subject, must not be forgotten: in the latter instance, they are found more or less collapsed, and the veins, which prior to death constituted but a small part of the swelling, are, both by their colour and size, the first objects that arrest the attention. The whole of this section is well written, and deserves the perusal of the young practitioner.

We pass over the sixth and seventh sections, which treat of hemorrhoidal varices, and the effects of inflammation of the common tumor, or piles, as they are usually called, since they contain nothing but what is generally known and admitted as true. With regard to the causes of this disease, more difference of opinion has been entertained. All the common opinions upon this point are respectively detailed by our author; and here we observe that, though he is intimately acquainted with all the authorities of the case, he is not prepared with any decided opinions of his own, as to whether the male or female is most subject to the disease; whether it be owing to hereditary predisposition, to climate, improper diet, or, lastly, to an habitually costive state of the body: and we conceive he is particularly unfortunate in deciding against this latter condition as exciting cause, since we have ever found it the most active and foremost amongst them. This condition of the system induces long sitting at the water-closet, perpetual strainings to evacuate the contents of the rectum, and, if long continued, is invariably followed by an increase of the symptoms. Those who, from circumstances or situation, are frequently obliged to postpone the discharge of the fæces, are also liable to attacks of piles; and the disease, when once formed, undoubtedly has a tendency to increase progressively. In middle life it is extremely common, both with males and females; though, if we were to decide by the result of our own experience, we should say that the hemorrhoidal tumors are most frequent in females who have been married, and borne large families; but that, *cæteris paribus*, they cause more uneasiness and distress when they affect the male sex, to which, in all probability, habits of life, more active employment, and in some measure the structure of the parts, may contribute. Many of the minor causes mentioned by our author, no doubt, also have a share in the formation of this disease: indeed, it appears to us to be one of those complaints in which numerous causes are generally combining, in the same individual, to the development of the symptoms.

In the ninth section, Mr. Calvert discusses the general treatment of hemorrhoidal tumors, commencing with some remarks on the notion formerly entertained, that this disease was in some respects salutary, and that to check it was a practice fraught with danger. Our author seems not quite to have made up his mind as to what degree of credit may be due to these often-repeated assertions; and he quotes a case from Mr. Howship, and mentions one which we suppose came under his own cognizance, of serious disease following the suppression of the discharge from these tumors. In Mr. Howship's case, death followed, from gout in the stomach, in three days. The other case does not appear to have been fatal. Such events are, however, rare, and, in guiding us in practice, can have little or no influence; since, when these tumors have acquired a great size, and are consequently interfering completely with the comfort of our patient's life, we do not hesitate to recommend their removal; and since, in an hundred such instances, the health will be found to be restored by the operation, our apprehensions of the ill consequences resulting from the cure or removal of the complaint in less severe cases, must amount to but a trifle indeed. Nevertheless, if there be decided gout in the habit, it may be well to take that circumstance into consideration; since the most trifling operation, the sudden application of cold, or any minor cause, may, in some constitutions, produce a fatal result; but we do not believe it to be peculiar to the treatment of piles.

We quote the following sensible directions for the management of the common attacks of piles, with great pleasure:—

"As the disposition to hemorrhoids, when promoted by circumstances, often continues for life, and the paroxysms are liable to be increased in number, or aggravated by numerous and very trifling causes, it becomes a heavy tax and inconvenience for the patient to have recourse, on every exigency, to medical opinion. For these reasons, it is in some degree incumbent on the practitioner to furnish his patient with such general rules as may tend to lessen the predisposition, and moderate the violence of the attacks, without risk. These should relate to the ordinary actions of the day, to diet, the state of the bowels, the circumstances under which topical applications are most useful, &c. &c.

"Attention to diet is the first and most important consideration in many cases; and, if the patient is not advanced in years, enjoys a moderate state of health, and will submit to what is thought requisite, I will venture to say that he may pretty confidently expect a perfect cure, with little foreign assistance.'

"It is not possible to give general directions respecting diet, that are applicable to every case, as much will depend upon peculiarity of constitution, the state of the general health, &c.; but, in general, that which consists of a mixture of animal and vegetable food, which contains sufficient nutriment in a moderate compass, is easily digested, and not too stimulating, is the best. In this respect, the patient must have recourse to that which he has found by experience to agree with him, and which, if possible, without the aid of medicine, will maintain the bowels in a state of proper action. Milk has been recommended, and where it agrees it certainly forms an excellent diet; but it is apt to confine the bowels, and form very hardened fæces, and the necessity, in such cases, of giving laxatives must counterbalance the virtues it may possess. I am at present, however, acquainted with an elderly lady, who for many years has suffered from hemorrhoids, and an excessively torpid state of the bowels, and in whom milk acts as a purgative. She is in the habit of taking a glassful, warm from the cow, every second morning, and prefers it to purgative medicines, as its operation is sufficiently powerful, without being accompanied with any griping pain or nausea.

"Those who suffer from hemorrhoidal diseases are often in the habit of taking purgatives, either to obviate costiveness, or to lessen the heat of the body, as it is vulgarly termed. No habit, however, can be more pernicious, particularly when these are of the resinous kind, which is generally the case, as they are more readily formed into pills. By purging, the contents of the bowels, being expelled before they have undergone the natural change, act upon the mucous membrane of the large intestines, like many foreign substances, to whose mode of excitement it is not habituated. At the anus, where there is more animal sensibility, this irritation is shown in the burning sensation that accompanies the expulsion of the fæces under such circumstances, and sometimes produces a degree of tenderness almost amounting to excoriation.

"Another habit, which is also very pernicious to those who are affected with hemorrhoids, is that of continually taking hot liquids. These gradually relax the vigorous tone of the stomach and bowels, render the whole system much more sensitive, and therefore more liable to be acted upon by change of temperature, and many other causes to which we are constantly exposed. They should, therefore, be taken very sparingly; and, where the previous habits of the patient have not rendered this temporary stimulus in some degree necessary, and the circumstances of the case may require a particular attention, it is much better that they should be altogether withdrawn. I have had repeated opportunities of witnessing the good effects of a moderated diet and cool drinks; but those who have been accustomed to take large quantities of hot tea, coffee, &c., and depend upon them for that pleasurable state of mind which is natural to some without their influence, can seldom be prevailed upon to forego their use.

"What I have said respecting hot liquids refers also to the frequent use of hot stimulating injections; a practice which is less necessary to notice, because it is not common in this country. The same objections, however, do not obtain to cold washes and injections, which, if used with prudence, are always of the greatest service whenever hemorrhoidal diseases are not owing to a torpid state of the circulation. Indeed, injections of cold water have been so strongly recommended by Schmucker and some other celebrated continental writers, that they are now in pretty general use." (P. 73—80.)

As a remedy for obviating the costive state of the body, our author praises the supertartrite of potash combined with sulphur; and attributes, with great propriety, the benefit derived from Ward's paste, cubebs, or other stimulating remedies, to their employment in those cases of long standing proceeding from debility, or a sluggish state of the circulation.

This section concludes with directions for our conduct when the sudden suppression of an attack of piles is succeeded by severe constitutional symptoms.

The local treatment of hemorrhoidal tumors, which in fact comprises their radical cure, resolves itself into three modes,—the application of medicinal substances, compression, or finally their removal with the knife or ligature. To fulfil the first intention, the assiduous employment of lotions and ointments is recommended; and, where the tumor is either single or small, and so situated as to be within the reach of these applications, we have known the decoction of oak-bark especially productive of the greatest good. Our author says, that these washes are more efficacious if combined with pressure,—which we admit; but we have not found that patients in general are inclined to submit to this inconvenience in cases where the tumors are not, from their size or number, a perpetual source of uneasiness; and then the more active measure of removing them alone holds out a prospect of affording permanent relief. When the tumor is more internal, the use of the rectum bougie will frequently be productive of great benefit. All these measures, however, are adapted to the treatment of piles in their quiescent state: when, from some accidental circumstances, they become inflamed, they then must be treated according to the common principles of surgery. Rest, in the horizontal posture, is necessary; local bleeding we have always found to afford great relief; and, from experience, we are inclined greatly to prefer warm fomentations upon such occasions. Our author, however, recommends cold injections; though he admits that, from the tenderness of the parts, injections cannot often be had recourse to, without causing great pain. We again repeat, that warm water, or the poppy fomentation, will, in this condition of the parts, be most acceptable to the patient's feelings.

Mr. Calvert discusses at some length the propriety of applying leeches to piles under these circumstances, and notices the strong opposition made to this proposal by Richter and other eminent men: he seems himself not to be decided upon this point. For our own parts, we have done it so often ourselves, and with such decided advantage, that we have no hesitation in recommending it; and upon what principle it can be objected to, we are at a loss to conceive.

Our author remarks that, when the tumors cannot be returned, they very often swell and become livid; and, in fact, are destroyed in the same manner as if a ligature had been applied.

Very few words will suffice upon the subject of compression as a curative means in this class of diseases: however plausible it may appear in theory, it is scarcely practicable. Compression must be employed for a long time before any permanent good can be expected from it. It is incompatible with the usual avocations and pleasures of mankind, and therefore never will be submitted to in the majority of instances; it is also uncertain, to say the best of it, in its effects.

We therefore proceed to discuss the propriety of removing these tumors, by the knife or ligature. If we look at the conflicting opinions of eminent authors, both English and foreign, ancient and modern, we shall be able from them to form no conclusion whatever, as to the preference to be given to one mode of operating rather than another in these cases. Each has some melancholy story to tell of death from ligature or hemorrhage, as they happen to advocate one or other method: but the whole secret lies in a very narrow compass, and the line of distinction is well defined and certain. Whenever the hemorrhoidal tumor is so situated that *skin* is involved in its removal, the ligature should never be resorted to: it is in those instances that the alarming and fatal symptoms noticed by authors occur, and as, in such cases, the fear of hemorrhage cannot operate, there can be no objection to the use of the knife. Where, however, the disease is situated fairly within the rectum, and the tumors are merely covered by the mucous membrane of the gut, the ligature is the safest, indeed the only safe, method of removing them; for we have seen and known them to bleed in a very alarming manner when removed by the knife; they escape out of our reach, and we have no certain method of commanding the hemorrhage: whereas, when the ligature is employed, it is true that the pain is often great,—that the bladder sometimes sympathises with the rectum, so as to produce partial retention of urine, requiring the employment of the catheter, perhaps, for a day or two; and, more rarely, the stomach feels the impression of the operation, and there is nausea and sickness; but the pulse is rarely affected. The symptoms to be dreaded are *not those of inflammation*,—they are to be commanded by the free exhibition of opium; and we agree entirely with Mr. Howship, in believing that, in consequence of the action of the ligature, the parts previously relaxed become more firmly consolidated. The only precaution necessary to be taken in these cases is to evacuate the bowels well previously, to keep the patient in bed, to calm pain by the use of opium, and to prevent the parts being used for three or four days, if possible. If the tumors to be removed are large, it is best to pass a needle, armed with a double ligature, through the base, and to tie it on each side. If the attachment of the tumor be small, a single ligature will be sufficient. They should in both cases be drawn sufficiently tight.

We refer to Mr. Calvert's work, for a description of either mode of operating; but we are quite sure that the distinction we have made above is both a just and a safe one.

We have now arrived at the second division of Mr. Calvert's work, on *Stricture of the Rectum*, a complaint which is undoubtedly more common than it was formerly believed to be, but which has become, perhaps, too fashionable in some parts of the kingdom; since we have heard that all diseases are, in a certain quarter, referred without hesitation to this source. We must take this opportunity of saying, that we consider Mr. White's little work upon this disease as a very valuable addition to our stock of knowledge; and that we have only been prevented from noticing the second edition by a pressure of other matter.

To return to the work before us: we consider the chapter on Strictures of the Rectum as well drawn up, containing all the facts materially necessary to be known; and the practical instructions are also well detailed. It must be recollected, that both prolapsus ani and hemorrhoidal tumors may originate from a strictured state of the rectum; and that it is, therefore, necessary to make accurate inquiries into the symptoms in all such cases; and, if any doubt exists, to examine the part with a bougie. We extract from our author the following method of making this examination:—

"Whenever the symptoms lead to a suspicion that there is a contraction in some part of the rectum, it is absolutely necessary, in the first place, to ascertain if this be really the case, as well as its nature, if possible, situation, extent, &c. This can only be done by a scientific and careful examination of the gut; which is the more requisite, because nearly the same train of symptoms may proceed from a variety of causes, which will be afterwards noticed; and if purgatives be exhibited, or the bougie employed, without this precaution, the patient may be exposed to considerable risk, and the cause of his sufferings increased instead of remedied, by the means that are carelessly adopted for its removal. For this purpose, the rectum being cleared out by a common clyster, and the patient placed upon his side, or (what is better) resting upon his knees and elbows, the finger of the surgeon should be smeared with common cerate, and carefully introduced within the gut. If no contraction be discovered, it may be presumed that one exists higher up; and a common plaster-bougie, rendered somewhat pliant by warmth, and slightly bent near the end, so as to accord, in some degree, with the natural curvature of the passage, must be anointed in a similar manner, and gradually passed onwards, if no obstacle intervene, within the sigmoid flexure of the colon. This operation requires some caution and judgment on the part of the surgeon; for, if it is not performed with delicacy, the parietes of the gut may possibly be injured, or, the upper axis of the pelvis being overlooked, the point of the bougie may be directed against the projecting part of the sacrum, and give rise to the idea of a stricture, when none in reality exists. At the same time, also, it is useful to bear in mind, that a similar mistake may possibly occur when the upper part of the gut, being distended with fæces, is forced down, and in some degree turned upon itself; or the cavity may be almost obliterated by the pressure of tumors, as in cases of enlarged ovaria, retroversion of the uterus, &c." (P. 165—167.)

"With reference to the length of time the bougie should be allowed to remain within the rectum, it must be regulated solely by the feelings of the patient, or rather by the manner in which the act of dilating the stricture may affect the constitution; for he may suffer severely from this cause, without being at all aware that his symptoms can be attributed to it. If the pressure of the bougie, although moderate, cause considerable pain in the situation of the stricture, extending to the groins, the thighs, or other parts; or if, after the bougie is withdrawn, general uneasiness, tremors, and sickness come on, we may conclude that, in the present state of the patient at least, it will do more harm than good; and the common tent, or the dilated gut, should be substituted. As these symptoms, however, may proceed from violence in using the bougie, in cases where, if judiciously employed, it might be of essential service, it should not be discontinued, when found to disagree, without first trying one of a smaller size, and taking care to introduce it with delicacy and judgment. Indeed, in employing pressure in any form for the cure of strictures of the rectum, it should always be recollected that the disease is in general produced and kept up by local irritation, and that violence of any kind is more likely to increase than remedy the evil. The surgeon should, therefore, be supplied with a number of bougies of different sizes and consistence; and the first that is used should be just large enough to produce a very moderate degree of distention. This may be withdrawn after remaining a few minutes, if it produce much pain or uneasiness; the time being gradually increased afterwards, as the part becomes habituated to the pressure. The size of the instrument must also be gradually increased, in proportion as the stricture is distended, until at last one of the largest diameter can be introduced, and retained with ease." (P. 177—179.)

This division of our author's work also contains directions for the treatment of the cancerous stricture, as well as for other varieties of the common stricture, and which require occasionally the use of the knife; but want of space obliges us to pass hastily over the remaining portion of this chapter, simply remarking, that it is very creditable to Mr. Calvert's talents, and well deserving perusal.

The third chapter, on *morbid contraction of the anus*, leads our author, after the detail of a severe case of this kind, to notice, and to doubt, the opinion advanced by DELPECH, and other foreign writers, of the occasional venereal origin of this complaint. If mercury, either locally or generally applied, be useful in these cases, it is scarcely warrantable, merely on that account, to decide upon it as a symptom of syphilis; added to which, mercury alone, without the employment of other means, will not be found to effect a cure.

In the history of the spasmodic contraction of the anus, arising frequently from fissures within the gut, our author gives us the result of M. Boyer's opinions and practice, as detailed by Mr. White, and in some of the late periodical publications; but he seems to doubt whether the operation recommended by that gentleman is so often called for, or so often likely to be successful, as it was found to be in M. Boyer's hands. We shall give his reasons in his own words:—

"In the first place, the necessity in the operation of including the fissure in the division seems to imply, either that the contraction is consequent to the fissure, or that the violent paroxysms of pain are produced solely by the efforts to dilate the sphincters, when morbidly contracted; an opinion that arises from the circumstances of the pain following the evacuation of the fæces. In some cases, however, the paroxysms of pain and contraction occur periodically; and in others, the sufferings of the patient are much less violent, but more continued, particularly when the complaint proceeds from sympathy with other parts; yet both these cases may exist without the presence of fissure.

"From this view of the complaint, it would appear that the distention of the anus, in evacuating the fæces, is amongst the number of those causes that aggravate the morbid sensibility already existent in the sphincter muscles; and that, in dividing these, we merely contribute to the cure in removing one accidental source of irritation, whilst the disposition to the complaint, and other causes by which it has been produced, or at least is kept up, still remain.

"Although this form of contracted anus is often connected with fissures, yet, as it sometimes exists separately, it is evident that these, when present, cannot, with any degree of certainty, be considered as the cause, and that we cannot conclude the complaint will disappear, if these are cured. In cases of piles, excrescences, and other affections of the anus, connected with thickening and disordered state of the internal membrane, fissures are very common; owing, I conceive, to the greater disposition to the cracking and excoriation of this part in passing hardened fæces, and the difficulty with which cicatrization is effected, where the adjacent parts are liable to frequent motion: and we may, therefore, expect to find them, with much more reason, whenever the sphincters (as in the present lamentable complaint) are so rigidly contracted, that the inner membrane of the anus must necessarily become thickened, and often excoriated, from a similar cause. Still, however, they may have existed previously, and have given rise, either separately or in conjunction with other causes of irritation, to this painful affection of the sphincter muscles; and whenever, therefore, from the urgency of the symptoms, the operation is determined upon, the instrument should always be carried through the principal fissure, unless it is situate at the anterior part of the anus; for, even admitting that it is a secondary affection, still, as the constant irritation it must necessarily produce may tend to increase the original complaint, the operation, if unsuccessful, would not be wholly useless." (P. 228—230.)

He suggests also, theoretically, (for he has had no opportunity of trying it in practice,) that, as this complaint usually occurs in persons of a weak and irritable habit of body, it may sometimes be considered as a species of tic douloureux; and that, therefore, when the digestive organs are in some degree restored to a healthy action, the sulphate of quina, or carbonate of iron, may be useful.

We shall pass entirely over the fourth chapter, on *prolapsus ani*, and its mode of treatment; since, although it contains much matter, there is nothing that requires comment or criticism. It gives a full and fair account of the complaint.

Of *fistula in ano*, and the treatment of *abscesses near the anus*, our author gives an extended account; but which the small portion of room that we can afford to bestow upon the subject, obliges us merely to notice. Mr. Calvert teaches us how much may sometimes be achieved by proper management of these abscesses. He warns us not to be too hasty in our use of the knife in recent cases; and he very properly cautions us against interfering too hastily with those fistulæ evidently connected with a disordered state of the constitution. In these instances it will, of course, be more likely to succeed, if the original disorder be removed, or even palliated. He then proceeds to describe two modes of performing the operation, by the knife and by the ligature. In the former case, our author avails himself of the judicious remarks of Dr. Ribes, and adds this important advice:

"In performing the operation for fistula in ano, no pains should ever be spared in endeavouring to discover the internal aperture. This, by some surgeons, is not considered of much importance; but, having seen the operation fail from neglect of this kind in two or three instances, I am inclined to think otherwise. The sinus, it is true, will heal to the whole extent; but a small aperture, from which a little matter continues to exude, remains within the gut: and, as this is prevented from healing with facility, in consequence of the motion of these parts, and of the irritation of the moisture that oozes from the fæces, another sinus may form, requiring the operation to be repeated. For this reason, I think it advisable to discover the internal aperture, and, when the sinus happens to extend still higher, that it should, if possible, be included in the division." (P. 311.)

We shall not add any account of the mode of operating by the ligature. Our author does not altogether condemn it; at least, he seems to think it may occasionally be warrantable. We hardly think so, but are not willing to enter into a controversy upon this point; since, indeed, Mr. Calvert himself does not insist very strongly upon it.

A short chapter on *ulcers of the rectum*, and another on *excrescences about the anus*, conclude the volume. Concerning these, little need be said: their causes and mode of treatment are pretty well understood, and fortunately in this country we have not often to deal with those of a specific nature, excepting—But the subject is invidious and disgusting, and therefore we will omit even the exception.

We must now draw to a close. We take our leave of Mr. Calvert, by thanking him for the pleasure we have derived from the perusal of his work. We have not concealed our honest opinion, that it is too long, too much crowded with quotation, and occasionally not definite enough in its practical directions; but yet it contains undoubted evidence of his industry, his literary attainments, and his professional zeal.

### DIVISION II.

### FOREIGN.

ART. III.—*De Medulla Spinali Nervisque ex ea producentibus Annotationes Anatomico-Physiologicæ.* Auctore CAROLO FRANCISCO BELLINGERI, Regiæ Scientiarum Academiæ et Collegii Medici Taurinensis Membro, Imp. et Reg. Scientiarum, Litterarum, et Artium Academiæ Batavinæ Sodali, Regiæ domus Medico.—Augustæ Taurinorum ex Tygographia Regia. 1823.

*Anatomical and Physiological Remarks on the Spinal Marrow, and the Nerves arising therefrom.* By CHARLES FRANCIS BELLINGERI, &c. &c.

So general an attention has recently been excited, both at home and abroad, towards the anatomy and functions of the nervous system, that no apology can be necessary for renewing the subject; particularly as we have made it our business to lay before our readers the earliest information of every novelty on this subject, and as this Journal has been the medium of several highly interesting and important original communications.* The work before us relates more especially to the spinal marrow, a portion of the human frame, which, although possessed of the highest physiological interest, has, from its peculiar situation, been less examined than other parts; the care with which nature has guarded it from injury, rendering it at the same time less easily exposed for the purpose of anatomical scrutiny. Accordingly, there are few parts, the descriptions of which have differed more essentially, with respect to circumstances about which no discrepancies could, *a priori*, have been anticipated: thus, in speaking of the distribution of the grey matter in the centre of the white, LIEUTAUD says that it has a form of two half-moons; WINSLOW compares it to a horse-shoe; HUBER to the os hyoides; MONRO says it resembles a cross; HALLER a double cross *(quadricruris)*; while others, particularly GALL and M. BELLINGERI, differ in their account of it from all these.

The last mentioned is the writer now before us, who informs us that he has for some time directed his investigations towards the spinal marrow, and paid particular attention to the relative distribution of the white and grey substances. In doing this, he followed the method recommended by RACCHETTI, of hardening the cord by means of nitric acid, much diluted with water; or, what answers still better, the fuming nitrous acid, although, in using this, considerable care is required not to employ the acid in too large proportion, lest it stain the whole yellow, so that

---

* See various papers on the Nerves, by Mr. SHAW and Mr. BROUGHTON, published during the last two years.

the different structures cannot be distinguished from each other. The water, we are informed, is sufficiently acidulated when it communicates a distinctly sour taste to the tongue. Thus prepared, it may be kept for months or years, and its structure clearly demonstrated; but, if it be cut across in its recent state, particularly if the weather be at all hot, the substances are so soft as to be semi-fluid; a circumstance which renders it difficult to ascertain its internal arrangement, and which sufficiently accounts for the contradictory descriptions of it above mentioned.

M. Bellingeri proceeds to treat, at considerable length, of the distribution of the grey matter in man and other animals, and subsequently describes the external appearance and the origin of the nerves. Instead, however, of following him through this detail, we shall lay before our readers the conclusions at which he arrives; in doing which, we shall follow the text as closely as is consistent with perspicuity.

The summary, then, of M. Bellingeri's opinions is, that the spinal marrow is composed of two substances,—viz. grey and white; the former being situated in the centre, the latter on the surface; that its structure is fibrous; that the quantity of the white substance in the whole spinal cord is much greater than the quantity of the grey, except in the sacral region, where the proportions are either equal or in favour of the grey. The form of this last generally resembles the figure )( ; the anterior horns of the grey matter no where reaches so far as the periphery of the cord: and these phenomena are presented by the spinal marrow of the human species, the ox, kid, and birds. The grey substance in man is placed rather anteriorly as far as the lumbar region, where, however, as well as throughout the whole region of the sacrum, it is either in the middle, or inclines towards the posterior surface. But, in the spinal cords of the other animals above mentioned, it is generally placed rather to the posterior part, except in the region of the second and third cervical vertebræ in the ox, and the middle of the lumbar region, and the upper and middle parts of the sacral region, where it either occupies the centre or inclines forwards; in birds, it is much more anterior in the middle of the sacral region. In man, and the other animals above mentioned, the portion of the grey substance is constantly different in the superior lumbar and superior sacral regions, from what it is in other parts. The nervous filaments arise from both of these substances, white and grey.

There are in the human subject, and in the animals above mentioned, central furrows, anterior and posterior; whereof the former is the larger, but no where penetrates so far as the grey substance; the second is smaller, and descends till it touches the said substance. There are, besides, in all spinal cords, lateral furrows, anterior and posterior, extending through the whole length of the cord. From the disposition of these grooves and fissures, in conjunction with the arrangement of the grey matter, it arises that the spinal marrow in man, and the other animals above mentioned, is divided into six fasciculi, of which two are anterior, two lateral, and two posterior. The anterior fasciculi are nearly, but not entirely, separated from each other: in the same way, they are divided from the lateral; but the posterior are entirely detached, both from each other and from the lateral. This arrangement is common to man and animals. The lateral fasciculi are almost every where thicker and more crowded than the rest, and they give to the spinal cord its greater or less degree of thickness. The posterior fasciculi in man (except in the lumbar and sacral regions) are thicker than the anterior: on the contrary, in the other animals above alluded to, the anterior fasciculi are generally rather thicker than the posterior, except in the superior cervical region in the ox, and in the inferior lumbar and sacral regions in the ox, kid, and birds. The anterior fasciculi, as well as the posterior, are generally of a flat form, and are alternately increased and diminished in thickness in various parts of their course. The anterior fasciculi communicate, in man, with the corpora pyramidalia, the crura cerebri, and thence with the brain proper; the lateral fasciculi are continued into the corpora restiformia; while the posterior fasciculi communicate directly with the cerebellum. So that it appears, as stated by Soemmering, that the spinal cord is chiefly composed of prolongations from the cerebellum; or, at least, communicate chiefly with it.

With regard to the structure of the anterior roots of the spinal nerves, it is demonstrated that they are composed of filaments, having a triple origin,—first, from the anterior fasciculi; secondly, from the region of the anterior lateral fissures; and lastly, from the lateral fasciculi. There are, however, some fibres which rise directly from the white substance of the anterior and lateral fasciculi; others which, perhaps, come from the anterior horns of the grey matter; and lastly, there are some which come from the surface of the spinal marrow, and others from its interior. The anterior roots are composed of numerous nervous fibres, having nearly the same thickness, each of which arises separately, approaches the neighbouring fibres of the same root, but does not blend with them: each fibre is about the size of a hair, and is never subdivided into smaller threads. The anterior roots, at least in man, do not enter the spinal ganglia.

The posterior roots are composed of fibres, rising in a triple order: the chief part comes directly from the posterior cornua of the grey substance; other filaments, but these are few in number, have their origin from the white substance of the posterior fasciculi; others, in a similar manner, from the lateral fasciculi. The second and third sets of fibres come from the surface and deeper parts of the substance of the cord. There are filaments, however, which are finer than the others, being of the same size as the fibres of the anterior roots: these are not numerous. In another set, the filaments are much thicker and more numerous, being composed of many very small fibres, which are much interwoven, both with each other and with the neighbouring filaments, so that the fibres of the posterior root can by no means be separated; and thus these roots and their filaments, both in their origin and course, present a plexuose form. Frequently the neighbouring posterior roots of the same side communicate with each other by means of nervous twigs; and these roots alone constitute the spinal ganglia.

By instituting a comparison between the anterior and posterior roots, four circumstances may be remarked, in which they differ:—1st. The filaments of the posterior roots are, generally speaking, thicker and less numerous than the filaments of the anterior roots; 2d, the filaments of the posterior alone present a plexuose structure; 3d, the posterior roots alone form the spinal ganglia; 4th, the neighbouring posterior roots almost all communicate by nervous filaments, [illegible]

All the filaments of the accessory nerves arise from the lateral fasciculi of the spinal cord, and penetrate deep into its substance: no communication intervenes between the accessory nerve and the posterior roots of the spinal nerves. Such is the case in the ox; but, in the human subject, although the nerve has the same origin, it sometimes receives many or all the fibres of the posterior roots of the first pair; and occasionally, although but seldom, a filament of the same root of the second pair. But the accessory nerves do not retain these fibres, which, on the contrary, go to constitute or increase the posterior root of the first pair of cervical nerves.

*Of the functions of the anterior roots.*—That the anterior roots of the spinal nerves are composed of filaments having a triple origin, has been anatomically demonstrated, as well as that some of these come from the anterior fasciculi, some from the region of the anterior lateral fissures, and others from the lateral fasciculi. Now, M. Bellingeri regards those filaments which come from the anterior fasciculi as serving the purpose of voluntary motion, because they arise from prolongations of the cerebrum; and holds that anatomy, physiology, and pathology, combine to prove that the brain proper presides over voluntary motion: thus differing in opinion from his colleague,

ROLANDO, from FLOURENS, and other recent experimentalists. The grounds assigned for his dissenting are as follow:—The third pair, or motor oculorum, which is a nerve of motion alone, rises chiefly from the crura cerebri; the sixth pair, or abductor oculorum, likewise purely a nerve of motion, arises from the superior extremity of the corpora pyramidalia, which again are productions of the cerebrum; the principal fibres of the twelfth nerve, or hypoglossus, which presides over the motions of the tongue, likewise arise from the external sides of the corpora pyramidalia. So much for anatomy and physiology, as proving that the brain proper regulates voluntary motion; while, with regard to pathology, he remarks, that paralysis, occurring on the side opposite the injury received, proves the brain proper to be the seat of motion; because, if hemiplegia occurred from injury of the cerebellum, it could not be on the opposite side, from the absence of any decussations of its prolongations. He further supports himself by the authorities of FRANCK and WILLIS; and thus, holding it to be demonstrated that the cerebrum, and its prolongations, govern voluntary motion, he thinks it reasonable to suppose that the filaments of the anterior roots, arising from the anterior fasciculi of the spinal marrow, are likewise destined for the same purpose. With respect to the filaments of the anterior roots which have their origin in the region of the anterior lateral fissures, he hazards no conjecture; seeing he is doubtful whether they arise from the white matter only, or partly from the grey likewise. But, with regard to the filaments coming from the lateral fasciculi, he holds them to be destined for the organic functions; an opinion which, he thinks, is rendered probable by the analogy of the spinal accessory. Certain filaments of this nerve rise from the lateral fasciculi of the cord, and form the internal branch; which branch, uniting with the gastro-pulmonary, can only perform organic and involuntary functions. He likewise believes the filaments of the anterior roots coming from the lateral fasciculi, to be those which concur to form the intercostal nerve; but this is advanced rather as a conjecture than as an assertion. It is not supposed, however, that all the filaments in question are expended upon the intercostal, but that many of them go to constitute part of the spinal nerves, and are thus distributed over the various parts of the body, performing the natural or organic functions,—as circulation, absorption, nutrition, secretion, &c.

*Of the uses of the posterior roots.*—Touching the uses of the posterior roots, the following opinions are advanced:—Those filaments which come from the posterior fasciculi, are thought to be destined for voluntary motion; those which come from the lateral fasciculi, are supposed to answer the same purposes as the corresponding filaments of the anterior roots,—viz. the regulation of the organic functions. The filaments arising from the posterior cornua of the grey matter, are supposed by our author to communicate the sense of touch. The posterior roots alone present a plexuose structure in their origin and course: they constitute the spinal ganglia, and are composed of thicker filaments than the anterior roots; besides, many of them communicate directly with the grey matter,—characters distinguishing the nerves of sensation from those of function. The olfactory nerves, the optic, and the auditory, evidently rise from grey matter, or communicate with it at their origin, (the first of these, indeed, is chiefly formed of this substance,) and have a plexuose or ganglioniform structure; besides which, they are larger than the nerves destined for the purposes of motion. On the other hand, the nerves of voluntary motion arise from the white medullary matter, have no plexuose or ganglioniform structure, and are thinner or finer than the sentient nerves; such are the third, fourth, and sixth, the fascial nerve, and the hypoglossus. Thus, according to M. Bellingeri, the posterior roots alone are destined to effect the sense of touch; and further, that this function is only performed by those filaments of the posterior roots which rise directly from the posterior cornua of the grey matter, or communicate directly with them. These opinions, he informs us, were committed to writing before he was made acquainted with the views recently advanced by other physiologists on this subject; and he dissents from those who hold that the posterior roots have no influence over motion.

In a chapter, "*De Nervorum Antagonismo*," we find some remarks upon the various kinds of motion performed by the human body, particularly with regard to the opposing actions of flexion and extension, adduction and abduction. Is it to be supposed (asks the author) that the nerves and nervous filaments which come from the brain, cerebellum, and their prolongations, serve indiscriminately for these movements? Are not the cerebral nerves rather destined for the one kind of motion, and those of the cerebellum for the other? These queries he answers, by declaring his belief that the nerves coming from the cerebrum, and its productions, serve for the purposes of flexion and abduction; while those of the cerebellum, and its productions, serve for the functions of extension and adduction. The fourth pair of nerves (he remarks) are opposed to the sixth; the former being adductors, and coming from a production of the cerebellum; the latter abductors, and coming from a production of the cerebrum. The third pair governs numerous and opposite movements of the eye, and accordingly it has a double origin,—one from the cerebrum, and the other from the cerebellum; for, besides the trunk of the third pair coming from the crura cerebri, there are accessory nerves, discovered by MALACARNE, which arise from the crura cerebelli. The tongue, likewise, which is gifted with various and opposite movements, is supplied with nerves having both a cerebral and cerebellic origin. Carrying on this idea, the posterior roots of the spinal nerves are regarded as destined for the purposes of extension. In man, the posterior fasciculi are thicker than the anterior, throughout almost the entire length of the spinal cord, which gives a corresponding superiority to the posterior roots; but, in the human body, the perpendicular position is more frequent than any other, and this position requires greater power of extension. In the upper part of the neck of the ox, the posterior fasciculi are much thicker than the anterior fasciculi, which is accounted for by the force required to sustain the head. But, in the region of the back, the posterior fasciculi are not so thick as the anterior; the horizontal position of the animal requiring less power in the dorsal muscles. Upon the same principle, it may be explained why the posterior fasciculi are much thicker than the anterior in the sacral region alone of birds: viz. the exterior muscles of the lower extremities requiring greater strength to support the body when the animal stands. The author next proceeds to support this view of the subject by pathological references, particularly to tetanic affections; but into these we cannot afford space to follow him.

We have a few words on the uses of the grey and white matters, the object of which, as above mentioned, is to show that the former is intended for sensation, and the latter for motion; and a chapter on the *spinal accessory*; but this contains nothing of interest to those acquainted with the recent discoveries on this subject.

---

